# Association between atherogenic index of plasma and microvascular complications in patients with type 2 diabetes achieving low-density lipoprotein cholesterol targets: a real-world cohort study

**DOI:** 10.7189/jogh.16.04350

**Published:** 2026-10-23

**Authors:** Shengchao Zhang, Yuzhao Shao, Min Li, Yuhan Wang, Hanbing Yu, Richang Cong, Zhenyuan Chen, Xiaoxv Yin, Ying Zhou, Huanhuan Ren

**Affiliations:** 1 Baoan Central Hospital of Shenzhen, Shenzhen, Guangdong, China; 2 Department of Social Medicine and Health Management, School of Public Health, Tongji Medical College, Huazhong University of Science and Technology, Wuhan, Hubei, China

**Keywords:** type 2 diabetes, low-density lipoprotein cholesterol, atherogenic index of plasma, microvascular complications

## Abstract

**Background:**

Despite achieving low-density lipoprotein cholesterol (LDL-C) targets, patients with type 2 diabetes mellitus (T2DM) remain at significant risk for microvascular complications. We aimed to investigate whether the atherogenic index of plasma (AIP), a marker of atherogenic dyslipidaemia, is associated with these complications in a real-world cohort of patients with controlled LDL-C.

**Methods:**

We conducted a retrospective cohort study using electronic health records from community health centres in Shenzhen, China (2018–2024). We included 16,469 participants with T2DM and LDL-C<2.6 mmol/L at baseline, categorising them into tertiles (T1–3) based on baseline AIP. The primary outcome was a composite of incident diabetic retinopathy, diabetic nephropathy, and diabetic peripheral neuropathy, identified *via* ICD-10 codes. We used multivariable Cox proportional hazards models to assess associations.

**Results:**

During a median follow-up of 4.6 years, 1,056 participants developed microvascular complications. We observed a graded association between AIP tertiles and the risk of microvascular complications. Participants in T3 had a significantly higher risk of total microvascular complications than those in T1 (hazard ratio (HR) = 1.34; 95% confidence interval (CI) = 1.15–1.56), while T2 showed no statistically significant increase in risk (HR = 1.10; 95% CI = 0.94–1.28). Specifically, compared to T1, T3 participants had significantly higher risks of diabetic nephropathy (HR = 1.62; 95% CI = 1.30–2.01) and diabetic peripheral neuropathy (HR = 1.39; 95% CI = 1.13–1.70), while no statistically significant association was found for diabetic retinopathy risk (HR = 1.23; 95% CI = 0.93–1.63). In contrast, compared with T1 participants, those in T2 had higher risks of nephropathy (HR = 1.28; 95% CI = 1.02–1.59), but no statistically significant associations were found for diabetic peripheral neuropathy or diabetic retinopathy.

**Conclusions:**

We found that AIP is independently associated with increased risk of microvascular complications, particularly diabetic nephropathy and diabetic peripheral neuropathy, even in T2DM patients achieving LDL-C targets. These results suggest that AIP may serve as a simple, lipid-based marker to help identify patients with residual microvascular risk beyond conventional LDL-C control.

The global burden of type 2 diabetes mellitus (T2DM) continues to rise rapidly, with 589 million adults affected worldwide and 148 million in China alone [1]. Among patients with T2DM, microvascular complications such as diabetic retinopathy (DR), diabetic nephropathy (DN), and diabetic peripheral neuropathy (DPN) represent major causes of morbidity and reduced quality of life [2].

Low-density lipoprotein cholesterol (LDL-C) remains the primary lipid target in T2DM management, with guidelines recommending levels <2.6 mmol/L [3,4]. However, notable residual risk of microvascular complications may still exist even after LDL-C targets are achieved [5–8]. This suggests that LDL-C alone may not fully capture microvascular risk and highlights the need for additional lipid-related markers.

The atherogenic index of plasma (AIP), calculated as log(triglycerides (TG)/high-density lipoprotein cholesterol (HDL-C)), reflects both the quantity and quality of atherogenic lipoproteins and is closely linked to insulin resistance and inflammation [9–11]. Previous studies have shown that elevated AIP is associated with increased risk of cardiovascular outcomes in the general T2DM population, with some evidence also suggesting a link to microvascular complications [10–14]. However, evidence regarding the association of AIP with microvascular complications specifically among patients who have already achieved LDL-C targets remains limited. Evaluating AIP in this population is of public health relevance, as it may help identify individuals with residual microvascular risk beyond conventional LDL-C control in real-world settings.

Therefore, we aimed to investigate the association between AIP and the risk of incident microvascular complications among patients with T2DM who achieved LDL-C targets (<2.6 mmol/L), using a large real-world community-based cohort in Shenzhen, China.

## METHODS

### Study design and participants

China's National Basic Public Health Service Standards mandate the creation and maintenance of electronic health records for individuals with T2DM at the community level, along with annual comprehensive health examinations. We conducted a retrospective cohort study using de-identified electronic health records from community health service centres in Shenzhen, China (2018–2024).

Participants were patients with a physician-diagnosed T2DM, as recorded in the community diabetes health management database according to the Chinese Diabetes Society guidelines and ICD-10 codes (E11). We included individuals with a confirmed diagnosis of T2DM, available baseline lipid measurements (*i.e.* LDL-C, TG, and HDL-C), and an LDL-C treatment target (<2.6 mmol/L) at baseline. We applied no strict age restrictions, as a broad age spectrum better reflects the real-world community diabetes population. We calculated follow-up time from the baseline examination date to the date of hospitalisation for microvascular complications or the study end date. Because outcome ascertainment relied on inpatient records, we imposed no minimum follow-up period and included all eligible participants regardless of follow-up duration. We excluded participants with pre-existing microvascular complications at baseline [12], LDL-C≥2.6 mmol/L at baseline [4], or missing baseline HDL-C or TG data.

The baseline database initially included 66,926 patients with T2DM in Shenzhen from 2018 to 2024 (**Figure 1**). After applying the eligibility criteria, we included 16,469 participants in the final analysis.

**Figure 1:**
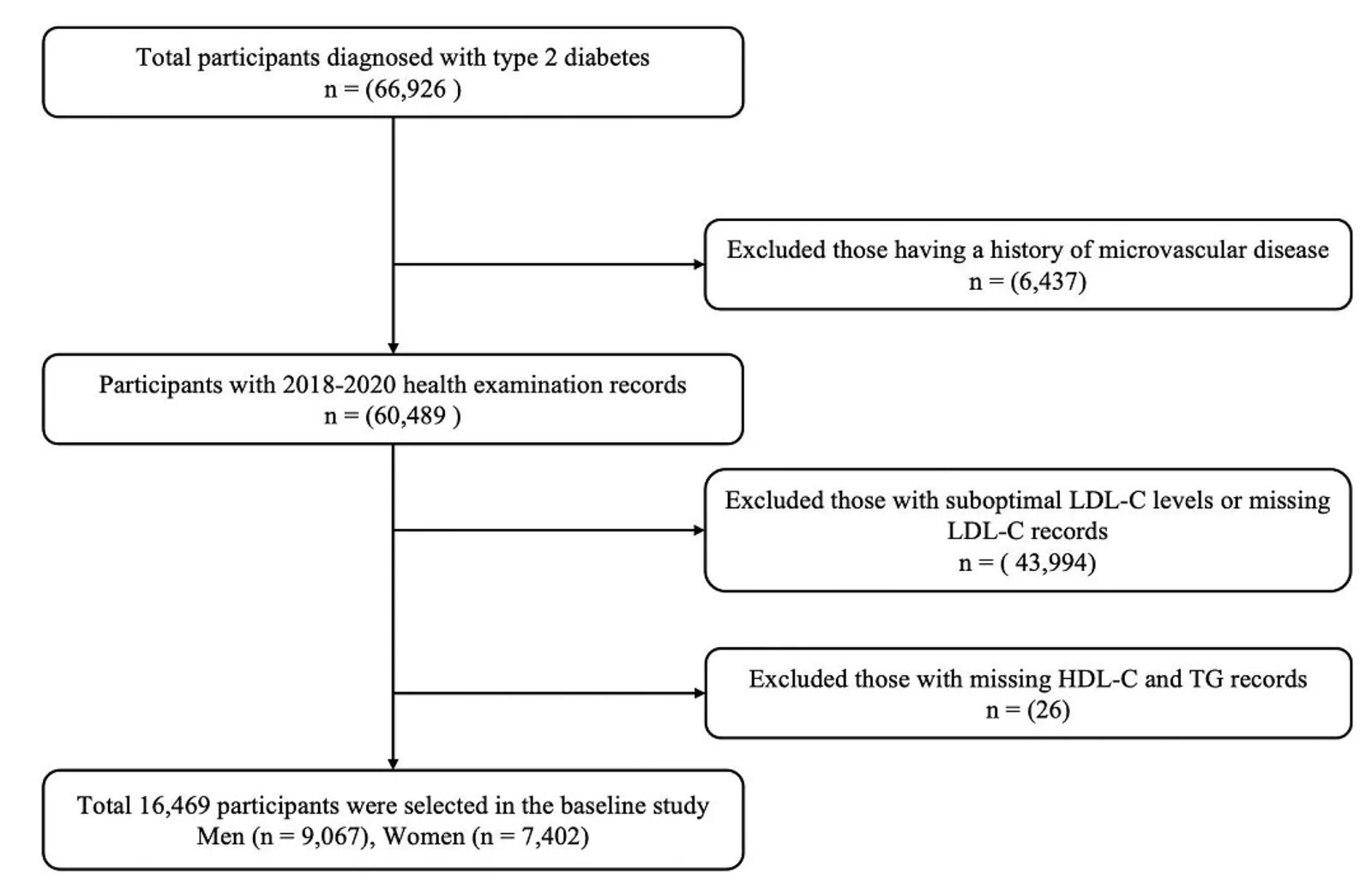
Study population selection. LDL-C – low-density lipoprotein cholesterol, HDL-C – high-density lipoprotein cholesterol, TG – triglycerides.

### Assessment of AIP

The AIP is a logarithmically transformed ratio of TG to HDL-C in molar concentration (mmol/L), and it is mathematically derived from log(TG/HDL-C) [9]. Nurses collected venous blood samples for laboratory testing, including measurements of TG, HDL-C, LDL-C, and other relevant parameters. We then classified the study population into three groups by AIP tertile: T1 (≤−0.43), T2 (−0.43–0.24), and T3 (>0.24).

### Ascertainment of outcome

Microvascular complications served as the primary outcome for the cohort. The secondary outcomes included DR, DN, and DPN [15]. We extracted complication data from inpatient medical records. We identified cases using ICD-10 diagnostic codes for DR (E11.3, E14.3, H28.0 and H36.0), DN (E11.2, E14.2, N18.0, N18.1, N18.2, N18.3, N18.4, N18.5, N18.8 and N18.9), and DPN (E11.4, E14.4, G59.0, G62.9, G63.2 and G99.0).

### Covariates

For this study, we derived covariate data from three sources: the diabetes baseline database, the annual health examination database, and electronic prescription records. Trained physicians or nurses collected baseline and annual health examination data through laboratory tests and structured interviews, while medication information was ascertained by matching drug names in electronic prescription records. We defined covariates as a set of potential confounders encompassing demographic, physical examination, lifestyle, and medical history factors. These included sex (male, female), age (year), educational level (primary school or below, middle school, high school or above) [16], body mass index (BMI), smoking status (never, former, current), drinking status (none, mild, moderate, heavy) [17], regular exercise (yes, no), duration of diabetes (>5 and ≤5 years), diagnosis of hypertension (yes, no), family history of diabetes (yes, no), use of oral antidiabetic drugs (yes, no), use of insulin (yes, no), and use of statins (yes, no). We calculated BMI as weight (kg) divided by height squared (m^2^) [18]. Heavy drinkers were defined as those who consumed alcohol every day. In contrast, self-reported occasional drinkers and frequent drinkers were defined as mild and moderate drinkers, respectively [17]. Trained physicians or nurses collected smoking status, drinking status, and regular exercise through face-to-face structured interviews during health examinations, in accordance with the standardised protocols of China's National Basic Public Health Service Standards. We recorded all interview data directly into the electronic health record system. We ascertained medication use from electronic prescription records in the community health database. For each participant, we extracted all medication records during the observation period and classified them into three categories based on drug name matching: oral antidiabetic drugs (including but not limited to metformin, sulfonylureas, DPP-4 inhibitors, SGLT-2 inhibitors, and thiazolidinediones), insulin preparations, and statins (including atorvastatin, rosuvastatin, simvastatin, and other lipid-lowering agents of the statin class). We considered a participant a user of a given medication category if the corresponding drug was prescribed at least once during the observation period. We included these medication variables as binary covariates (yes/no) in the multivariable-adjusted models. Hypertension was defined as a self-reported diagnosis, or an average systolic blood pressure ≥140 mm Hg, or an average diastolic blood pressure ≥90 mm Hg [19], or the administration of antihypertensive medication. Family history of diabetes was defined as at least one parent, sibling, or child with diabetes [20].

### Statistical analysis

Baseline characteristics of participants were described according to AIP tertiles [21,22]. We presented normally distributed continuous variables as mean (standard deviation) and compared them between groups using one-way analysis of variance. We presented non-normally distributed continuous variables as median (interquartile range) and compared them using the Kruskal-Wallis H test. Lastly, we presented categorical variables as frequencies (percentages) and compared them using the χ^2^ test [23].

Missing data were examined for all covariates (Table S1 in the **Online Supplementary Document**). In the primary analysis, we performed multiple imputation by chained equations using the ‘mice’ package in *R*. We generated five imputed datasets with ten iterations each, using a fixed random seed to ensure reproducibility. Pooled estimates were obtained using Rubin’s rules.

To evaluate the association between AIP levels and the incidence of microvascular complications, we constructed multivariable Cox proportional hazards regression models to estimate hazard ratios (HRs) and 95% confidence intervals (CIs) for each group. The proportional hazards assumption was verified using Schoenfeld’s residual test, and no violation was detected. We defined three models: Model 1 (unadjusted), Model 2 (adjusted for age, sex, and education level), and Model 3 (further adjusted for smoking status, drinking status, regular exercise, BMI, duration of diabetes, family history of diabetes, hypertension, use of oral antidiabetic drugs, use of insulin, and use of statins). To assess the dose-response association, AIP was entered into the Cox models as a continuous variable. We presented the results as HRs per one-unit increase in AIP using the three adjustment models.

We then performed subgroup and interaction analyses. We analysed the association between AIP and microvascular complications within predefined subgroups, including age, sex, BMI, and duration of diabetes. We also introduced interaction terms to test for effect modification.

To verify the robustness of the findings, we conducted sensitivity analyses. First, to address potential bias from multiple imputation, we excluded individuals with missing covariate values and conducted a complete-case analysis. Second, we excluded participants using oral antidiabetic drugs, insulin, or statins; Third, we additionally adjusted for total cholesterol and LDL-C levels in Model 3. Finally, we excluded participants developing microvascular complications within one year of follow-up to minimise potential reverse causality.

We performed all statistical analyses using *R*, version 4.4.3 (R Core Team, Vienna, Austria). All *P*-values were two-tailed, and a *P*-value <0.05 was considered statistically significant.

## RESULTS

### Baseline characteristics

We included 16,469 participants. The median age was 57.0 years (interquartile range = 48.0–66.0), and 9067 (55.1%) were male. Missing data for key variables were generally low, including diabetes duration (2.59%), drinking status (0.56%), smoking status (0.37%), regular exercise (0.15%), BMI (0.12%), and lipid profiles (<0.3%). No missing data were present for the remaining covariates. Based on AIP tertiles, participants were categorised into three groups: T1 (n = 5,490), T2 (n = 5,491), and T3 (n = 5,488). Overall, individuals with higher AIP levels were more likely to be male, possess higher educational attainment, exhibit higher BMI, engage in heavy alcohol consumption and current smoking, while demonstrating lower rates of regular exercise (**Table 1**). Furthermore, higher AIP levels were associated with significantly higher rates of hypertension, family history of diabetes, use of oral antidiabetic medications, and statin therapy.

**Table 1:** Characteristics of the study participants by AIP tertiles, n (%)

	Overall (n = 16,469)	T1 (n = 5,490)	T2 (n = 5,491)	T3 (n = 5,488)	*P*-value
Age in years*	57.0 (48.0, 66.0)	58.0 (49.0, 67.0)	58.0 (49.0, 67.0)	55.0 (46.0, 65.0)	<0.001
**Sex**					<0.001
Male	9,067 (55.1)	2,874 (52.3)	2,912 (53.0)	3,281 (59.8)	
Female	7,402 (44.9)	2,616 (47.7)	2,579 (47.0)	2,207 (40.2)	
**Education**					<0.001
Primary school or below	5,549 (33.7)	1,884 (34.3)	1,937 (35.3)	1,728 (31.5)	
Middle school	5,747 (34.9)	1,928 (35.1)	1,830 (33.3)	1,989 (36.2)	
High school or above	5,173 (31.4)	1,678 (30.6)	1,724 (31.4)	1,771 (32.3)	
BMI (kg/m^2^)*	24.3 (22.4, 26.6)	23.4 (21.5, 25.5)	24.4 (22.5, 26.7)	25.2 (23.3, 27.3)	<0.001
LDL-C (mmol/L)*	2.1 (1.7, 2.4)	2.0 (1.6, 2.3)	2.1 (1.7, 2.4)	2.1 (1.7, 2.4)	<0.001
HDL-C (mmol/L)*	1.2 (1.0, 1.4)	1.4 (1.2, 1.7)	1.2 (1.0, 1.3)	1.0 (0.9, 1.2)	<0.001
TC (mmol/L)*	4.2 (3.6, 4.7)	4.1 (3.6, 4.6)	4.0 (3.5, 4.6)	4.4 (3.8, 5.0)	<0.001
TG (mmol/L)*	1.5 (1.0, 2.4)	0.9 (0.7, 1.1)	1.4 (1.2, 1.7)	3.0 (2.2, 4.4)	<0.001
**Duration of diabetes in years**					<0.001
≤5	11,148 (67.7)	3,595 (65.5)	3,667 (66.8)	3,886 (70.8)	
>5	5,321 (32.3)	1,895 (34.5)	1,824 (33.2)	1,602 (29.2)	
**Hypertension**					<0.001
No	6,668 (40.5)	2,528 (46.0)	2,097 (38.2)	2,043 (37.2)	
Yes	9,801 (59.5)	2,962 (54.0)	3,394 (61.8)	3,445 (62.8)	
**Family history of diabetes**					<0.001
No	13,534 (82.2)	4,631 (84.4)	4,508 (82.1)	4,395 (80.1)	
Yes	2,935 (17.8)	859 (15.6)	983 (17.9)	1,093 (19.9)	
**Drinking status**					<0.001
None	13,866 (84.2)	4,736 (86.3)	4,671 (85.1)	4,459 (81.2)	
Mild	1,517 (9.2)	445 (8.1)	471 (8.6)	601 (11.0)	
Moderate	453 (2.8)	115 (2.1)	156 (2.8)	182 (3.3)	
Heavy	633 (3.8)	194 (3.5)	193 (3.5)	246 (4.5)	
**Smoking status**					<0.001
Never	12,854 (78.0)	4,492 (81.8)	4,345 (79.1)	4,017 (73.2)	
Ever	1167 (7.1)	390 (7.1)	416 (7.6)	361 (6.6)	
Current	2,448 (14.9)	608 (11.1)	730 (13.3)	1,110 (20.2)	
**Weekly exercise**					<0.001
No	5,089 (30.9)	1,561 (28.4)	1,622 (29.5)	1,906 (34.7)	
Yes	11,380 (69.1)	3,929 (71.6)	3,869 (70.5)	3,582 (65.3)	
**Use of oral antidiabetic drugs**					<0.001
No	1,999 (12.1)	800 (14.6)	625 (11.4)	574 (10.5)	
Yes	14,470 (87.9)	4,690 (85.4)	4,866 (88.6)	4,914 (89.5)	
**Use of insulin**					<0.001
No	14,129 (85.8)	4,602 (83.8)	4,736 (86.3)	4,791 (87.3)	
Yes	2,340 (14.2)	888 (16.2)	755 (13.7)	697 (12.7)	
**Use of statin**					<0.001
No	11,613 (70.5)	4,057 (73.9)	3,793 (69.1)	3,763 (68.6)	
Yes	4,856 (29.5)	1,433 (26.1)	1,698 (30.9)	1,725 (31.4)	

AIP – atherogenic index of plasma, BMI – body mass index, HDL-C – high-density lipoprotein cholesterol, LDL-C – low-density lipoprotein cholesterol, T – tertile, TC – total cholesterol, TG – triglycerides

*Presented as median (interquartile range) unless specified otherwise.

### Association between AIP and the risk of diabetic microvascular complications

During a median follow-up of 4.6 years, 1,056 participants developed microvascular complications, including 595 (3.6%) DPN cases, 535 (3.2%) DN cases, and 330 (2.0%) DR cases (**Figure 2**, Panel A–D). The incidence density (per 1000 person-years) was 7.9 for DPN, 7.1 for DN, and 4.4 for DR (**Table 2**). In addition, the higher AIP groups showed a significantly increased cumulative incidence of total microvascular complications over the follow-up period after the baseline survey (log-rank test *P* < 0.05).

**Figure 2:**
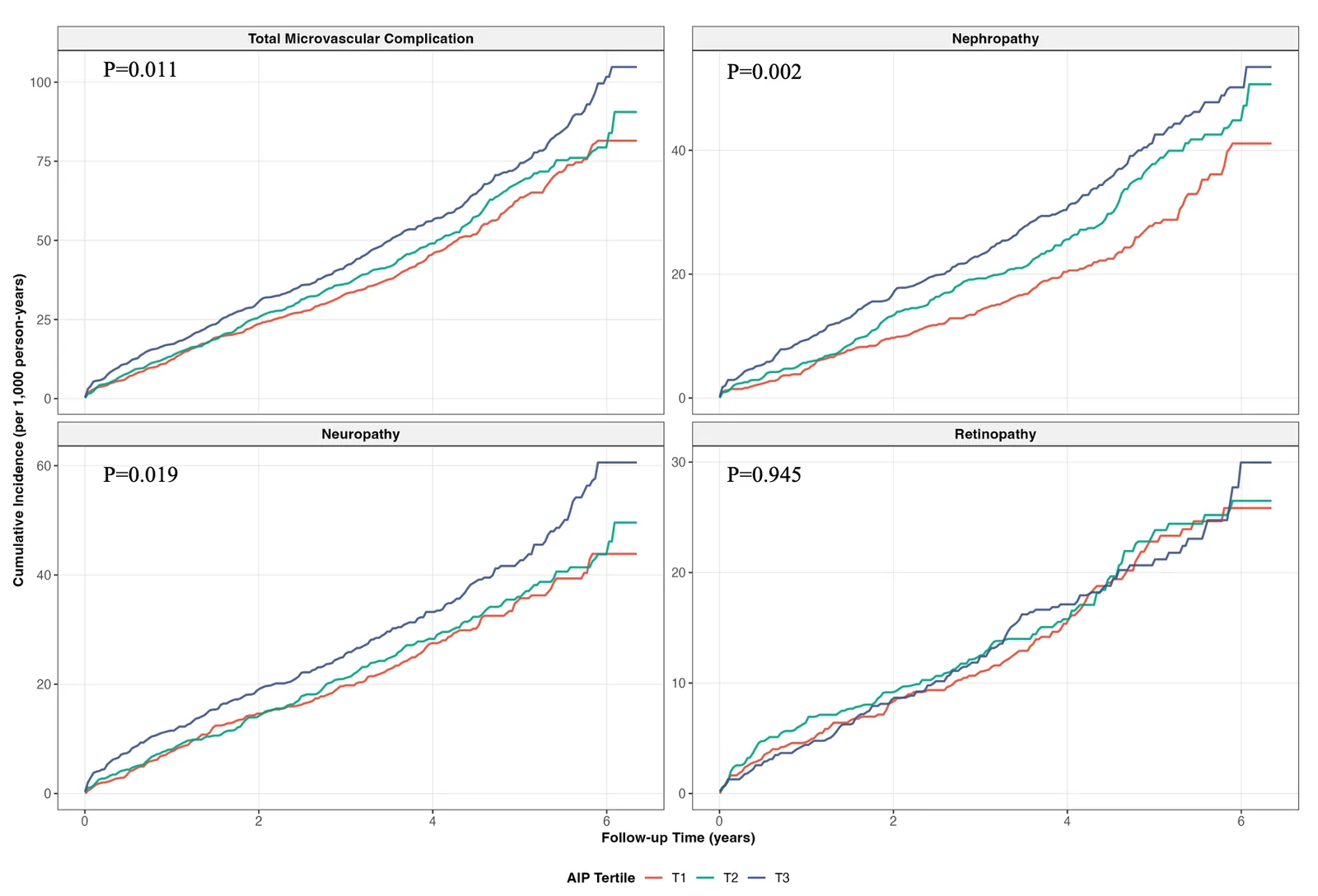
Kaplan-Meier curves for microvascular complications by AIP tertile. **Panel A.** Total microvascular complication. **Panel B.** Nephropathy. **Panel C.** Neuropathy. **Panel D.** Retinopathy. AIP – atherogenic index of plasma.

**Table 2:** Incidence density of diabetic complications (per 1000 person-years)

	Cases/n	Person-years	DPN	DN	DR
**Overall**	1,056/16,469	75,554.4	595 (7.9)	535 (7.1)	330 (4.4)
**T1**	432/5,490	25,344.6	179 (7.1)	145 (5.7)	108 (4.3)
**T2**	481/5,491	25,249.7	188 (7.4)	182 (7.2)	111 (4.4)
**T3**	547/5,488	24,960.1	228 (9.1)	208 (8.3)	111 (4.4)

DN – diabetic nephropathy, DPN – diabetic peripheral neuropathy, DR – diabetic retinopathy, T – tertile

We performed Cox proportional hazards regression analyses to evaluate the association between AIP levels and the risk of diabetic microvascular complications. The proportional hazards assumption was satisfied (*P* = 0.58) (Table S2 in the **Online Supplementary Document**). The results showed that participants in T3 had a significantly higher risk of total microvascular complications than those in T1 in Model 1 (HR = 1.24; 95% CI = 1.07–1.44), Model 2 (HR = 1.26; 95% CI = 1.08–1.46), and Model 3 (HR = 1.34; 95% CI = 1.15–1.56). On the contrary, T2 showed no statistically significant increase in risk across all models (*P* > 0.05) (**Table 3**). However, the *P* for trend was significant across all models (*P* for trend <0.05), supporting a dose-response relationship between increasing AIP levels and the hazard of total microvascular complications.

**Table 3:** Association between AIP tertiles and incidence of diabetic microvascular complications*

	Model 1		Model 2		Model 3	
	HR (95%CI)	*P*-value	HR (95%CI)	*P*-value	HR (95%CI)	*P*-value
**Total microvascular complications**						
T1	ref		ref		ref	
T2	1.07 (0.92–1.25)	0.371	1.07 (0.92–1.25)	0.378	1.10 (0.94–1.28)	0.242
T3	1.24 (1.07–1.44)	0.004	1.26 (1.08–1.46)	0.002	1.34 (1.15–1.56)	<0.001
*P*-value for trend		0.004		0.002		<0.001
**DN**						
T1	ref		ref		ref	
T2	1.26 (1.01–1.57)	0.038	1.27 (1.02–1.58)	0.030	1.28 (1.02–1.59)	0.031
T3	1.46 (1.18–1.81)	<0.001	1.55 (1.25–1.92)	<0.001	1.62 (1.30–2.01)	<0.001
*P*-value for trend		<0.001		<0.001		<0.001
**DPN**						
T1	ref		ref		ref	
T2	1.05 (0.86–1.29)	0.622	1.05 (0.86–1.29)	0.639	1.09 (0.89–1.34)	0.418
T3	1.30 (1.07–1.58)	0.01	1.28 (1.06–1.56)	0.013	1.39 (1.13–1.70)	0.002
*P*-value for trend		0.009		0.012		0.002
**DR**						
T1	ref		ref		ref	
T2	1.03 (0.79–1.34)	0.821	1.03 (0.79–1.34)	0.835	1.15 (0.88–1.50)	0.311
T3	1.05 (0.80–1.36)	0.742	0.99 (0.76–1.30)	0.967	1.23 (0.93–1.63)	0.142
*P*-value for trend		0.743		0.966		0.141

CI – confidence interval, DN – diabetic nephropathy, DPN – diabetic peripheral neuropathy, DR – diabetic retinopathy, HR – hazard ratio, ref – reference, T – tertile

*Model 1 was unadjusted; Model 2 was adjusted for age, sex, and education level; Model 3 was further adjusted for smoking status, drinking status, regular exercise, BMI, diabetes duration, family history of diabetes, hypertension, use of oral antidiabetic drugs, use of insulin, and use of statins.

Analyses for the secondary outcomes revealed a specific association between AIP levels and the risk of diabetic complications. The association was most pronounced for DN. Using T1 as the reference, T3 showed an increased DN risk in Model 1 (HR = 1.46; 95% CI = 1.18–1.81), Model 2 (HR = 1.55; 95% CI = 1.25–1.92), and Model 3 (HR = 1.62; 95% CI = 1.30–2.01), indicating that elevated exposure levels were consistently associated with increased DN risk. Trend tests further supported significant relationships across tertiles in all models (*P* for trend <0.001), suggesting that higher AIP is closely associated with elevated risk of microvascular complications. For DPN, significant associations were only evident in T3 across all models. In Model 3, T3 had an HR of 1.39 (95% CI = 1.13–1.70) compared to T1, with a significant trend (*P* for trend = 0.013). However, no statistically significant associations were detected between AIP levels and incident DR in any model, with the 95% CIs for all HRs including 1.0.

When AIP was modelled as a continuous variable, each one-unit increase was significantly associated with higher risk of DN (HR = 1.81; 95% CI = 1.42–2.31, *P* < 0.001), DPN (HR = 1.34; 95% CI = 1.07–1.69, *P* = 0.013), and the composite microvascular complications (HR = 1.37; 95% CI = 1.15–1.64, *P* < 0.001) after full adjustment (Table S3 in the **Online Supplementary Document**). For DR, the association was in the same direction but was not statistically significant (HR = 1.33; 95% CI = 0.97–1.81, *P* = 0.073).

### Subgroup and sensitivity analysis

To explore the association between AIP and diabetic microvascular complications in more detail, we categorised the study population based on age, sex, BMI, and duration of diabetes. We then performed stratified analyses to test for interactions and adjust for confounders within subgroups (Table S4 in the **Online Supplementary Document**). Notably, we observed a significant interaction between age and AIP in diabetic DPN risk (*P*_interaction_ = 0.009). Specifically, a high AIP index was more strongly associated with an elevated risk of DPN among people aged ≥65 years than those aged <65 years. However, we did not detect any interaction among other subgroups.

We conducted several sensitivity analyses to assess the robustness of the main findings (Table S5–8 in the **Online Supplementary Document**). The associations of AIP and microvascular complications remained consistent after excluding individuals with missing covariate values. This association also remained unchanged after removing glucose/lipid-lowering drug users at baseline. Additionally, adjusting for total cholesterol and LDL-C levels in Model 3 did not compromise the stability of the results. The results also persisted after excluding participants with follow-up durations of less than one year.

## DISCUSSION

Analysing 16,469 patients with T2DM in Shenzhen who achieved target LDL-C levels (<2.6 mmol/L), we found that elevated AIP levels remain significantly and positively associated with the risk of diabetic microvascular complications, particularly DPN and DN. However, elevated AIP levels were not associated with DR.

Several studies have explored the role of AIP in diabetic complications. A Chinese cross-sectional study of 3,278 patients undergoing coronary angiography showed that AIP is positively correlated with the risk of coronary artery disease in patients with T2DM [12]. In addition, the China Health and Retirement Longitudinal Study, which included 8,727 participants aged ≥45 years, demonstrated that high AIP levels are associated with an increased risk of stroke in individuals with prediabetes and diabetes [13]. Another secondary analysis based on data from the Action to Control Cardiovascular Risk in Diabetes trial, involving 10,251 patients with long-standing T2DM, indicated that AIP was independently associated with the risk of major adverse cardiovascular events in patients with T2DM [14]. However, previous research has paid insufficient attention to diabetic microvascular complications [10]. Among the few studies that have examined this association, findings have been inconsistent. Li *et al.* reported a positive correlation between AIP and microvascular complications in a general T2DM population [10], while others have found weaker or non-significant associations, particularly for DR [24,25]. We extend the existing evidence by focusing specifically on patients who have already achieved LDL-C targets, a population in which residual risk remains poorly characterised, and the association with AIP has been less well studied.

We found that the association between AIP and microvascular complications is highly organ-specific. After adjusting for confounders, higher AIP was associated with significantly increased risks of both DPN and DN, but not with DR. The continuous variable further supported the association between AIP and microvascular risk, consistent with the primary tertile-based findings. Several potential mechanisms may explain the observed association between AIP and microvascular complications. As one of the most severe complications of T2DM, DPN significantly increases the risks of ulcers, non-traumatic amputations, and foot infections [26]. Abnormal lipid levels can increase blood viscosity and promote microthrombus formation [27], while an inadequate blood supply to the nervous system can disrupt energy metabolism and impair signal transmission [28], particularly in patients with high AIP levels. Consistent with previous research, the risk of diabetic kidney disease remains high even when LDL-C levels are well-controlled [29]. Levels of TG and/or HDL-C are considered residual risk factors for DN [30]. The renal microvasculature appears particularly sensitive to lipid abnormalities, as triglyceride-rich lipoproteins can promote glomerular injury through lipotoxicity, inflammation, and oxidative stress [31,32]. In contrast, the link between lipid profiles and DR appears less robust [24]. The blood-retinal barrier may offer some protection against damage from plasma lipoproteins, and intraretinal lipid transport may play a more critical role in DR development than plasma lipid levels [25]. However, some studies have found a significant association between AIP levels and DR in patients with T2DM [33,34]. These studies focused on the general T2DM population, whereas our investigation specifically targeted the subgroup of patients who had achieved LDL-C targets. This distinction may account for the discrepant findings regarding DR. Therefore, further multicentre clinical investigations are warranted to clarify the association between AIP and DR across different risk strata, particularly in populations with controlled conventional risk factors.

This study also has certain limitations. First, as an observational study, it cannot establish causality. Although we performed extensive multivariable adjustment and multiple sensitivity analyses, we cannot entirely rule out residual confounding. Important potential sources include glycaemic control (*e.g.* HbA1c), which had high missing rates and was not systematically recorded in the community health database. In addition, this real-world data set largely lacked longitudinal lipid profiles. Nevertheless, we adjusted for a wide range of demographic, lifestyle, clinical, and medication-related confounders, and the main findings remained robust in multiple sensitivity analyses. Future studies with more comprehensive data collection are warranted to further minimise residual confounding. Second, we identified microvascular complications exclusively from inpatient medical records using ICD-10 codes. Since DR, DN, and DPN are frequently diagnosed and managed in outpatient settings, some milder or early-stage cases may have been missed. This could have resulted in under-ascertainment of outcomes, potentially underestimating the incidence rates observed in this cohort. Thus, the significant associations we observed are likely to be conservative estimates of the true effect. Furthermore, the study population consists solely of Chinese individuals from the Shenzhen region, and the generalisability of the findings requires validation in other ethnic groups. Finally, AIP was calculated from single-time point measurements, which may not capture the potential impact of longitudinal changes on risk.

## CONCLUSIONS

We found that AIP is independently associated with diabetic microvascular complications, particularly DN and DPN, even when LDL-C targets are met. Our results suggest that AIP may serve as a useful complementary marker for identifying residual risk in this population. Future studies are warranted to confirm its clinical utility and value in routine diabetes management.

## Additional material


Online Supplementary Document

## Data Availability

**Data availability:** The data sets and codes analysed in this study are available from the corresponding author upon reasonable request.
