# Supplementary material for: Association between atherogenic index of plasma and microvascular complications in patients with type 2 diabetes achieving low-density lipoprotein cholesterol targets: a real-world cohort study
Source: J Glob Health. 2026 Oct 23;16:04350. doi: 10.7189/jogh.16.04350 (PMC13693299; doi:10.7189/jogh.16.04350)
Supplement: Supplementary file 1 [file jogh-16-04350-s001.pdf]

Supplement to: Zhang S, Shao Y, Li M, Wang Y, Yu H, Cong R, Chen Z, Yin X, Zhou Y, Ren H. Association between atherogenic index of plasma and microvascular complications in patients with type 2 diabetes achieving low-density lipoprotein cholesterol targets: a real-world cohort study. J Glob Health. 2026;16:04350.

Table S1. Summary of missing data for covariates..........1

Table S2. Proportional Hazards Assumption Testing for the Multivariable Cox Model 1

Table S3. Association between AIP (as a continuous variable) and microvascular complications..........2

Table S4. Subgroup analysis results..........3

Table S5. Sensitivity analysis excluding individuals with missing covariate values..........5

Table S6. Sensitivity analysis excluding glucose/lipid-lowering drug users..........5

Table S7. Sensitivity analysis additionally adjusting TC and LDL-C levels in Model 3.........6

Table S8. Sensitivity analysis excluding events within a year..........6

Table S1. Summary of missing data for covariates

Variable	Missing (n)	Missing (%)
**Diabetes duration**	**426**	**2.59**
**Drinking status**	**93**	**0.56**
**Smoking status**	**61**	**0.37**
**TC**	**43**	**0.26**
**Regular exercise**	**24**	**0.15**
**BMI**	**20**	**0.12**
**Age**	**0**	**0%**
**Sex**	**0**	**0%**
**Education level**	**0**	**0%**
**Family history of diabetes**	**0**	**0%**
**Hypertension**	**0**	**0%**
**Use of oral antidiabetic drugs**	**0**	**0%**
**Use of insulin**	**0**	**0%**
**Use of statins**	**0**	**0%**

Abbreviations: BMI, body mass index; TC, total cholesterol.

Table S2. Proportional Hazards Assumption Testing for the Multivariable Cox Model

Variable	χ^2	df	*P* value
Global test	13.27	15	0.58
AIP	0.39	2	0.82
Age	0.48	1	0.49
Sex	0.27	1	0.60
Education	0.11	1	0.74
Smoking status	1.38	1	0.24
Drinking status	0.004	1	0.95
Weekly exercise	0.50	1	0.48
Diabetes duration	0.67	1	0.41
Family history	0.91	1	0.34
BMI	0.34	1	0.56
Hypertension	0.79	1	0.37
Use of oral antidiabetic drugs	0.85	1	0.36
Use of insulin	2.33	1	0.13
Use of statin	0.97	1	0.32

Abbreviations: AIP, atherogenic index of plasma; BMI, body mass index; df, degrees of freedom.
Proportional hazards assumption was tested using Schoenfeld residuals.

Table S3. Association between AIP (as a continuous variable) and microvascular complications

Variables	Model 1		Model 2		Model 3	
	HR (95%CI)	*P*	HR (95%CI)	*P*	HR (95%CI)	*P*
**Total microvascular complications**						
AIP (per 1-unit increase)	1.26 (1.07-1.48)	0.006	1.28 (1.08-1.52)	0.004	1.37 (1.15-1.64)	<0.001
**(A) Diabetic nephropathy**						
AIP (per 1-unit increase)	1.55 (1.24-1.95)	<0.001	1.73 (1.36-2.18)	<0.001	1.81 (1.42-2.31)	<0.001
**(B) Diabetic peripheral neuropathy**						
AIP (per 1-unit increase)	1.25 (1.01-1.56)	0.043	1.24 (0.99-1.54)	0.061	1.34 (1.07-1.69)	0.013
**(C) Diabetic retinopathy**						
AIP (per 1-unit increase)	1.12 (0.83-1.50)	0.472	1.03 (0.77-1.39)	0.827	1.33 (0.97-1.81)	0.073

Abbreviations: HR, hazard ratios; CI, confidence interval; other abbreviations as in Table 1.

Model 1: unadjusted.

Model 2: adjusted for age, sex, and education level.

Model 3: further adjusted for smoking status, drinking status, regular exercise, BMI, diabetes duration, family history of diabetes, hypertension, use of oral antidiabetic drugs, use of insulin, and use of statins.

Table S4. Subgroup analysis results

Subgroup	T1	T2		T3		*P* for interaction
		HR (95% CI)	*P*	HR (95% CI)	*P*	
**Total microvascular complications**						
Overall	Ref.	1.10 (0.94-1.28)	0.2416	1.34 (1.15-1.56)	<0.001	
Age						0.107
<65 years		1.09 (0.90-1.32)	0.3944	1.25 (1.04-1.52)	0.0199	
≥65 years	Ref.	1.11 (0.86-1.43)	0.424	1.54 (1.19-1.99)	0.0011	
Sex						0.416
Male		1.14 (0.93-1.39)	0.2098	1.47 (1.21-1.79)	<0.001	
Female	Ref.	1.03 (0.81-1.32)	0.792	1.16 (0.90-1.48)	0.259	
BMI						0.193
Normal		0.97 (0.79-1.20)	0.797	1.35 (1.09-1.67)	0.005	
Obesity	Ref.	1.21 (0.96-1.53)	0.102	1.33 (1.06-1.67)	0.013	
Diabetes Duration						0.277
<5 years		1.21 (0.97-1.51)	0.0937	1.32 (1.06-1.65)	0.012	
≥5 years	Ref.	1.00 (0.81-1.24)	0.998	1.38 (1.11-1.71)	0.004	
**(A) Diabetic nephropathy**						
Overall	Ref.	1.25 (1.00-1.56)	0.047	1.49 (1.19-1.87)	<0.001	
Age						0.759
<65 years		1.28 (0.95-1.72)	0.108	1.12 (0.92-1.36)	0.007	
≥65 years	Ref.	1.20 (0.87-1.67)	0.275	1.39 (0.98-1.96)	0.063	
Sex						0.159
Male		1.30 (0.99-1.70)	0.053	1.49 (1.13-1.96)	0.005	
Female	Ref.	1.15 (0.78-1.71)	0.484	1.61 (1.09-2.37)	0.016	
BMI						0.263
Normal	Ref.	1.24 (0.92-1.66)	0.158	1.71 (1.26-2.32)	<0.001	
Obesity		1.29 (0.93-1.81)	0.132	1.53 (1.11-2.11)	0.01	
Diabetes Duration						0.277
<5 years		1.31 (0.96-1.79)	0.092	1.35 (0.99-1.85)	0.06	
≥5 years	Ref.	1.25 (0.91-1.70)	0.166	1.97 (1.45-2.68)	<0.001	
**(B) Diabetic peripheral neuropathy**						
Overall	Ref.	1.06 (0.86-1.31)	0.579	1.23 (1.00-1.52)	0.048	
Age						0.009
<65 years	Ref.	1.08 (0.84-1.39)	0.538	1.08 (0.84-1.39)	0.569	

≥65 years		0.99 (0.68-1.44)	0.958	1.71 (1.19-2.45)	0.004	
Sex						0.139
Male		1.20 (0.92-1.58)	0.186	1.53 (1.16-2.00)	0.002	
Female	Ref.	0.88 (0.64-1.21)	0.438	0.90 (0.65-1.26)	0.541	
BMI						0.475
Normal	Ref.	0.92 (0.69-1.21)	0.533	1.22 (0.92-1.63)	0.168	
Obesity		1.31 (0.95-1.81)	0.098	1.57 (1.16-2.13)	0.004	
Diabetes Duration						0.547
<5 years		1.12 (0.83-1.51)	0.479	1.28 (0.96-1.72)	0.099	
≥5 years	Ref.	1.06 (0.79-1.41)	0.709	1.50 (1.13-2.00)	0.005	
**(C) Diabetic retinopathy**						
Overall	Ref.	1.11 (0.85-1.45)	0.462	1.04 (0.78-1.38)	0.789	
Age						0.449
<65 years		1.06 (0.77-1.45)	0.721	1.00 (0.73-1.39)	0.987	
≥65 years	Ref.	1.23 (0.73-2.09)	0.44	1.33 (0.76-2.34)	0.322	
Sex						0.651
Male		1.17 (0.83-1.67)	0.374	1.22 (0.85-1.76)	0.279	
Female	Ref.	1.01 (0.64-1.55)	0.951	0.87 (0.55-1.37)	0.539	
BMI						0.077
Normal	Ref.	1.18 (0.83-1.66)	0.355	1.38 (0.96-1.98)	0.084	
Obesity		1.02 (0.67-1.56)	0.928	0.97 (0.64-1.47)	0.882	
Diabetes Duration						0.578
<5 years		1.65 (1.09-2.50)	0.019	1.27 (0.811-1.99)	0.297	
≥5 years	Ref.	0.87 (0.61-1.25)	0.45	1.21 (0.85-1.73)	0.284	

Abbreviations: HR, hazard ratios; CI, confidence interval; BMI, body mass index.

Table S5. Sensitivity analysis excluding individuals with missing covariate values

Variables	Model 1		Model 2		Model 3	
	HR (95%CI)	*P*	HR (95%CI)	*P*	HR (95%CI)	*P*
T1	Ref.		Ref.		Ref.	
T2	1.04 (0.89-1.21)	0.610	1.04 (0.89-1.21)	0.607	1.07 (0.92-1.25)	0.393
T3	1.22 (1.05-1.41)	0.010	1.23 (1.06-1.43)	0.007	1.31 (1.13-1.53)	<0.001
P for trend		0.018		0.012		0.002

Model 1: unadjusted.

Model 2: adjusted for age, sex, and education level.

Model 3: further adjusted for smoking status, drinking status, regular exercise, BMI, diabetes duration, family history of diabetes, hypertension, use of oral antidiabetic drugs, use of insulin, and use of statins.

Table S6. Sensitivity analysis excluding glucose/lipid-lowering drug users

Variables	Model 1		Model 2		Model 3	
	HR (95%CI)	*P*	HR (95%CI)	*P*	HR (95%CI)	*P*
T1	Ref.		Ref.		Ref.	
T2	2.07 (0.68-6.33)	0.202	1.98 (0.65-6.07)	0.23	1.68 (0.54-5.20)	0.369
T3	3.63 (1.29-10.19)	0.014	4.20 (1.49-11.85)	0.007	3.39 (1.17-9.80)	0.024
P for trend		0.018		0.007		0.038

Model 1: unadjusted.

Model 2: adjusted for age, sex, and education level.

Model 3: further adjusted for smoking status, drinking status, regular exercise, BMI, diabetes duration, family history of diabetes, hypertension, use of oral antidiabetic drugs, use of insulin, and use of statins.

Table S7. Sensitivity analysis additionally adjusting TC and LDL-C levels in Model 3

Variables	Model 1		Model 2		Model 3	
	**HR (95%CI)**	***P***	**HR (95%CI)**	***P***	**HR (95%CI)**	***P***
T1	Ref.		Ref.		Ref.	
T2	1.07 (0.92-1.25)	0.371	1.07 (0.92-1.25)	0.37	1.10 (0.94-1.28)	0.236
T3	1.24 (1.07-1.44)	0.004	1.29 (1.11-1.50)	<0.001	1.34 (1.15-1.56)	<0.001
P for trend		0.006		<0.001		<0.001

Model 1: unadjusted.

Model 2: adjusted for age, sex, and education level.

Model 3: further adjusted for LDL-C, TC, smoking status, drinking status, regular exercise, BMI, diabetes duration, family history of diabetes, hypertension, use of oral antidiabetic drugs, use of insulin, and use of statins.

Table S8. Sensitivity analysis excluding events within a year

Variables	Model 1		Model 2		Model 3	
	**HR (95%CI)**	***P***	**HR (95%CI)**	***P***	**HR (95%CI)**	***P***
T1	Ref.		Ref.		Ref.	
T2	1.06 (0.89-1.26)	0.511	1.06 (0.89-1.26)	0.517	1.07 (0.90-1.27)	0.445
T3	1.20 (1.01-1.42)	0.033	1.21 (1.02-1.43)	0.026	1.27 (1.07-1.51)	0.007
P for trend		0.018		0.013		0.004

Model 1: unadjusted.

Model 2: adjusted for age, sex, and education level.

Model 3: further adjusted for smoking status, drinking status, regular exercise, BMI, diabetes duration, family history of diabetes, hypertension, use of oral antidiabetic drugs, use of insulin, and use of statins.
